# Parasitic Load, Hematological Parameters, and Trace Elements Accumulation in the Lesser Spotted Dogfish *Scyliorhinus canicula* from the Central Tyrrhenian Sea

**DOI:** 10.3390/biology11050663

**Published:** 2022-04-26

**Authors:** Francesca Romana Reinero, Concetta Milazzo, Marco Minervino, Cristian Marchio, Mariacristina Filice, Laura Bevacqua, Gianni Giglio, Francesco Luigi Leonetti, Primo Micarelli, Sandro Tripepi, Donatella Barca, Emilio Sperone

**Affiliations:** 1 Department of Biology, Ecology and Earth Sciences, University of Calabria, 87036 Rende, Italy; fr.reinero@gmail.com (F.R.R.); concetta.milazzo@unical.it (C.M.); marcominervino90@gmail.com (M.M.); cristian_marchio@alice.it (C.M.); mariacristina.filice@unical.it (M.F.); laura.bevacqua9@gmail.com (L.B.); gianni.giglio@unical.it (G.G.); francescoluigi.leonetti@unical.it (F.L.L.); sandro.tripepi@unical.it (S.T.); donatella.barca@unical.it (D.B.); 2 Sharks Studies Center—Scientific Institute, 58024 Massa Marittima, Italy; primo.micarelli@gmail.com

**Keywords:** parasitology, hematology, ecotoxicology, trace elements, Mediterranean, elasmobranch

## Abstract

**Simple Summary:**

The elasmobranchs, being at the apex of the marine food chain, act as final receptors of polluting elements that are regularly discharged into the sea and, therefore, usually are considered good bioindicators of environmental pollution. The main goal of this study was to describe helminthic communities, hematological parameters, and the concentrations of trace elements in vertebrae, skin, and liver for a population of lesser spotted dogfish from the Tyrrhenian Sea. Findings suggest that there is sometimes a correlation among these parameters and between them as well as environmental pollution.

**Abstract:**

Parasitological, hematological, and ecotoxicological analyses were carried out on a population of lesser spotted dogfish *Scyliorhinus canicula* from the central Mediterranean Sea. Parasitological analyses highlighted a poor helminthic community, highly dominated by a single taxon represented by the cestode *Nybelinia* sp. No differences in the parasitic load between females and males were observed. Hematological analyses showed that the number of leukocytes was significantly lower in the sharks that resulted in parasitism, and this could be due to the ability of some trace elements, such as arsenic, weakening the immune system and exposing animals to a higher risk of parasite infection, although further hematological and parasitological analyses are required on a larger number of samples. Trace elements analyses in the vertebrae, skin, and liver highlighted that the most abundant and potentially toxic elements were lead (Pb), arsenic (As), and cadmium (Cd). Other trace elements were also abundant, such as manganese (Mn), zinc (Zn), nickel (Ni), copper (Cu), and iron (Fe). Pb, As, and Mn showed the highest concentrations in vertebrae, while Cd, Cu, and Zn were the highest in the liver, probably due to their concentration in the prey items of the sharks; Fe and Ni showed the highest concentrations in the skin, due to their presence in the water column, especially along the coast where animals were collected. The concentration of some trace elements analyzed in the vertebrae decreased with the growth of the sharks. These results confirm that elasmobranchs, being predators at the apex of the marine food chain, act as final receptors for a series of polluting elements regularly discharged into the sea.

## 1. Introduction

The lesser spotted dogfish *Scyliorhinus canicula* (Linnaeus, 1758) is a shark species widely distributed in the Mediterranean Sea. Its habitat extends from the surface to the limits of the continental shelf [1,2]. This species has an important role in the trophic web of demersal fish communities, representing an important trophic link between invertebrates and larger predatory fish [3]. However, there is still little knowledge on few aspects of the biology and ecology of this species, especially in the Mediterranean [4]. For example, analyses of the composition and the dynamics of the lesser spotted dogfish helminthic communities have been conducted in most cases in northeastern Atlantic waters [5,6], and were completely absent from the Mediterranean until 2017, when Dallarès et al. [4] performed the first study on the composition and seasonal dynamics of the parasite communities of *S. canicula* and *Galeus melastomus* from the northwestern Mediterranean. At the same time, many biological and ecological aspects of this species have been largely analyzed, such as hematology [7,8,9,10], and the understanding of the pressures of marine pollution through the analysis of the bioaccumulation of trace elements in the target tissues [3,11,12,13]. Many trace elements are known as potential toxic elements [14], since some of them are not essential elements and could be toxic even at low concentrations such as Pb, Cd, and As, while others, being essential or partially essential elements, are toxic only beyond certain concentrations, such as Mn, Zn, Cu, Ni, and Fe. The side effects related to potential toxic elements are many and, in some cases, serious, since they are responsible for the alteration of the composition of the blood, for the lack of DNA synthesis, and for the alteration of some biological processes such as the slowdown in growth rates, the delay in reaching sexual maturity, and the reduction in fertility, all factors that in some way affect the reproduction and the survival of the species [15]. Furthermore, the sharks, being at the apex of the marine food chain, act as final receptors of polluting elements that are regularly discharged into the sea: for this reason, they could be considered valid bioindicators of environmental pollution. In addition, benthic or demersal sharks could be more exposed to the accumulation of these elements in their tissues, as they are present to a greater extent in sediments than in surface waters [16]. This study aimed to describe, for a Tyrrhenian population of *S. canicula*,

(1) the helminthic communities and epidemiological indices in the anatomical districts, including correlations between the parasitic load and sex of the sharks;
(2) the hematological parameters in the blood and their correlations with parasitic load;
(3) the concentrations of trace elements in target tissues (vertebrae, skin, and liver) and their differences in bioaccumulation related to sex, total length (TL), parasitic load and growth rates in vertebrae of these sharks.

## 2. Materials and Methods

### 2.1. Sampling Collection

**Figure 1:**
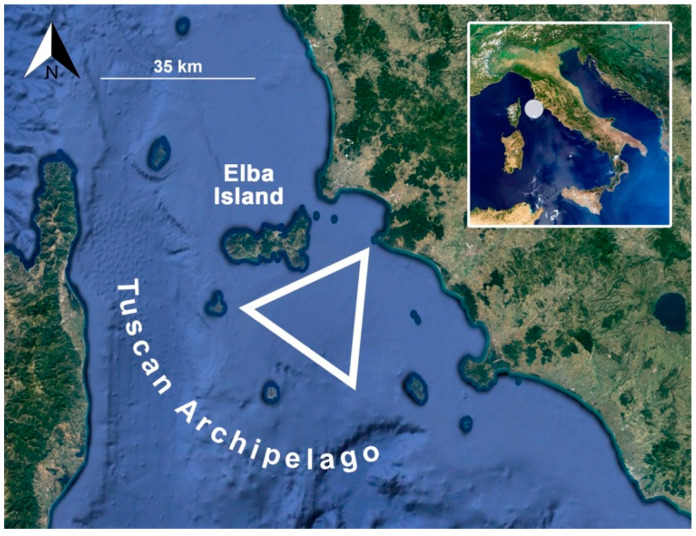
Localization of the sampling area. The white triangle indicates the fishing area of the samples.

The sampling was carried out at 12 nautical miles off Rocchette Punta Ala (Tuscany, Italy), between the Gulf of Follonica, Elba Island, and the Tuscan Archipelago (Figure 1), in collaboration with a local fishing boat “Mare Blu”. The sampling was carried out over two years and in four different months: February 2017, August 2017, January 2018, and May 2018. In total, 75 specimens of lesser spotted dogfish were collected by commercial bottom trawling at ca. 150 m depth and processed in the laboratory. An identification code was assigned to each sample before the dissection, and TL and weight were measured. The sex of each shark specimen was determined by the identification of claspers in males, and the maturity condition was confirmed by observing the presence of eggs and oocytes in females, as well as calcified claspers and sperm in male specimens [17]. Blood was drawn with a plastic syringe containing heparin from the caudal veins of 32 specimens. Each shark was then rinsed with fresh water and the possible presence of skin ectoparasites was observed. Finally, specimens were dissected at room temperature, and the organs necessary for parasitological and ecotoxicological analyses were removed and frozen at −20 °C.

### 2.2. Parasitological Analyses

The parasitological analysis was performed in different anatomical districts: mouth, gills, nostrils, dermal surface, liver, stomach, and spiral valve. The organs used for parasitological analyses were placed in Petri capsules containing 0.9 percent solution of sodium chloride to facilitate the identification of any external and internal parasites through the stereo microscope [18,19]. When present, parasites were isolated, photographed, counted for each sample examined, and placed in test tubes containing 70% ethyl alcohol solution to preserve them. Each parasite was mounted on a glass slide, with a single drop of Amman lactophenol (20% phenol crystalline, 40% glycerin, and 20% distilled water) placed carefully to clarify them, and their taxonomic characteristics useful for species identification were observed [20].

### 2.3. Hematological Analyses

The blood collected before dissection was transferred in 9 mL plastic tubes containing heparin to avoid coagulation, which were immediately placed at +4 °C. Samples were processed and analyzed using the DasitSysmex SX-1000i instrument, which made it possible to obtain the leukocyte formula of five populations using Fluorescence Flow Cytometry [21], the analysis of red blood cells and platelets with hydrodynamic focus, and the analysis of hemoglobin with the cyanide-free SLS method. In particular, for complete blood count, red blood cells (RCB), hemoglobin (HCB), average corpuscular volume of red blood cells (MCV), platelets (PLT), and white blood cells (WBC) were considered.

### 2.4. Ecotoxicological Analyses

**Figure 2:**
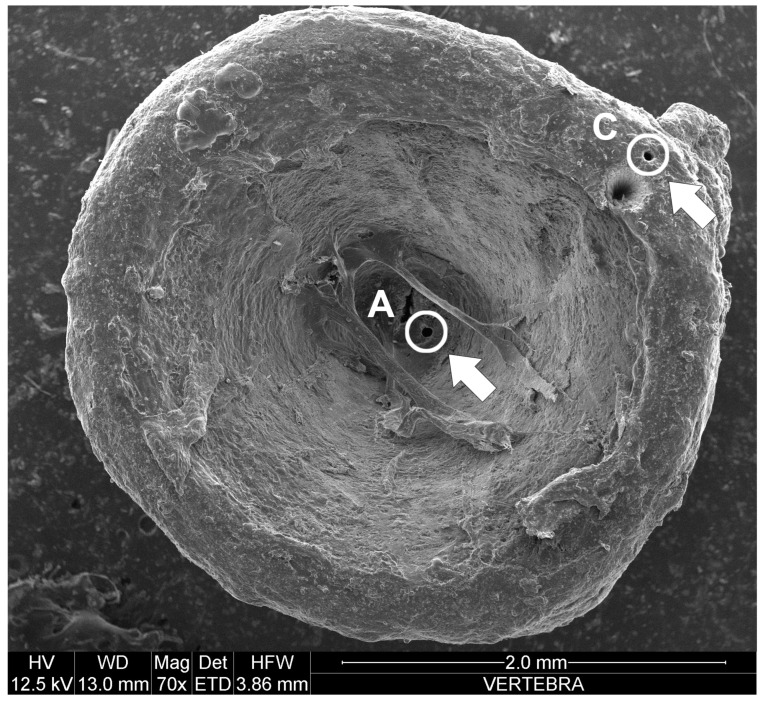
Vertebra of *S. canicula* showing the two perforations: one close to the center (point A) and one close to the distal part (point C), to analyze the concentration of trace elements in two different stages of the biological cycle of sharks. Circles and arrows indicate the positions of points A and C.

Analysis of the trace elements was performed for the vertebrae, skin, and liver. The use of vertebrae permits the analysis of trace elements in different stages of the shark’s life [22], given that the vertebrae grow by concentric rings during the growth of the individual. Therefore, the central rings will accumulate pollution information in the immature stages of the animals, while the marginal rings will accumulate information at the mature stages. In total, 75 vertebrae, one for each shark, were taken from the dorsal part of each sample close to the first dorsal fin. Each vertebra was cleaned of organic material, emal and neural arches, dried under a laminar flow fume hood at room temperature, and placed on a slide. Analyses were carried out using LA-ICP-MS (Laser Ablation Inductively Coupled Plasma Mass Spectrometer), an instrument used to determine almost all chemical elements (except liquid metals such as Hg) in solid samples [23,24]. The methodology made use of the combination of the New Wave Research UP213 solid-state Nd-YAG laser probe (213 nm) and Elan DRCe (Perkin Elmer/SCIEX) Inductively Coupled Plasma Mass Spectrometer (ICP-MS). Two perforations were performed with the laser for each vertebra: one close to the center (point A) and one close to the distal part (point C), to analyze the concentration of trace elements in ppm (μg/g) in two different stages of the biological cycle of sharks (Figure 2).

The laser beam was set up to perform perforations of 50 μm in diameter at a frequency of 10 Hz and at a fluence of ca. 20 J/cm^2^. The background levels of each element were established from the acquired data for ca. 60 s before ablation of the material, while the data acquisition took 60 s. The instrument’s external calibration was established using both a silicate glass material as reference (NIST SRM 612–650 ppm), produced by the National Institute of Standards and Technologies, and a USGS BCR 2G reference glass, as an unknown sample [25]. Calcium (^43^Ca) was determined by Electron MicroProbe (EMP) analysis and was used as an internal standard [26]. Data, acquired by the ICP-MS through the ELAN software, were finally processed through the GLITTER software. The accuracy, as well as the relative differences from the reference values, must always be less than 10%, with most of the elements included in a range of ±5%. For each measurement, three replicates were performed. Of the 46 trace elements analyzed by the instrument, only eight were selected (Pb, As, Mn, Zn, Ni, Cd, Cu, and Fe), considered as trace elements that could be potentially dangerous for the health of marine organisms [12] and because concentrations of the others were really low and, sometimes, undetected.

Regarding the analysis of trace elements in soft tissues, 12 skin samples and 12 liver samples were removed from 12 different sharks chosen from different classes of TL (providing a total of 24 analyzed samples). Samples were first dehydrated under the hood for 24 h at laminar flow without pulverization; afterward, they were weighed in Teflon containers on a high-precision analytical scale after its calibration, then placed in a solution of 10 mL of ultra-pure nitric acid (HNO_3_), digested for 50 min in the CEM MARS-5 microwave oven at 180 °C at a power of 400 W and at a pressure of 300 PSI, evaporated on a pre-heated plate at 200 °C under the hood, and, finally, brought to volume with ultrapure water in flasks of known volumes (50 or 100 mL depending on the weight of the sample) and stored in a refrigerator in containers at −4 °C. The same procedure was used to prepare Tort (lobster hepatopancreas) certified reference material (CRM), which was used as the unknown sample (for quality control standard) during the analytical sequence. After the acid attack, samples were analyzed through ICP-MS. When ready, all samples were introduced into the spectrometer through its peristaltic pump and transformed into an aerosol by the nebulizer supplied with the instrument. As for the vertebrae: of the 46 elements analyzed, eight were considered: Pb, As, Mn, Zn, Ni, Cd, Cu, and Fe.

### 2.5. Statistical Analyses

Parasitic infestation levels were calculated using the following epidemiological indices: prevalence (P%), average intensity (AI), and average abundance (AA) [27], while the correlation between the parasitic load and sexes was analyzed through the logit regression model using the R-Studio 1.1.423 program. Prevalence describes the fraction of the host population infected with a parasite. Intensity describes the average number of individual parasites of a given type present in an infected host. Finally, average abundance is the arithmetic mean of the number of individuals of a particular parasite species per host examined. All statistical analyses on hematology and ecotoxicology of sharks were carried out using the Instat 3.0 program. As for the hematological analyses, linear regressions were used to correlate the absolute values of the hematological parameters with each other and with biological parameters, and the Mann–Whitney test was used to verify any differences between the parasitic load of the sharks and the leukocytes number.

Regarding the ecotoxicological analyses in vertebrae, the concentrations of the eight elements considered were related to sex, TL, two different stages of the biological cycle of the sharks, and to the parasitic load using linear regressions, and the Mann–Whitney test was used to verify significant differences and relationships. Trace element concentrations found in the skin and liver were compared with those found in the vertebrae by means of the Kruskal–Wallis test.

## 3. Results

**Figure 3:**
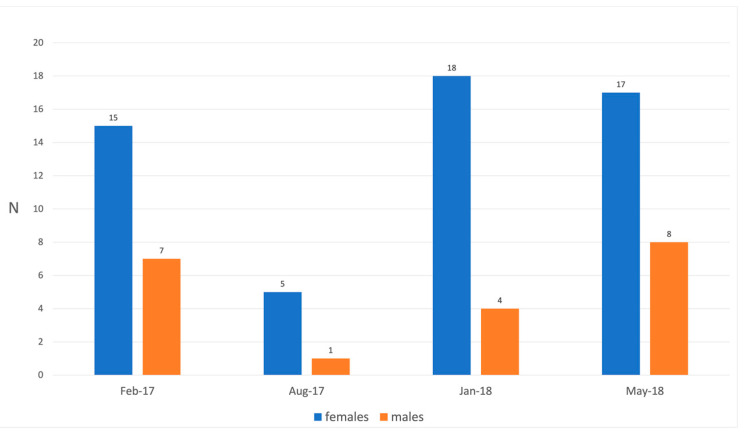
Frequency histogram of the numbers of specimens collected by month and by sex.

Of the 75 specimens of *S. canicula* sampled between 2017 and 2018 in four different months (February 2017, August 2017, January 2018, and May 2018), 55 were females (73%) and 20 were males (27%) (Table 1); the average length and weight of all the specimens and their seasonal distribution are reported in Table 1 and Figure 3, respectively. All specimens were sexually mature.

### 3.1. Parasitology

**Figure 4:**
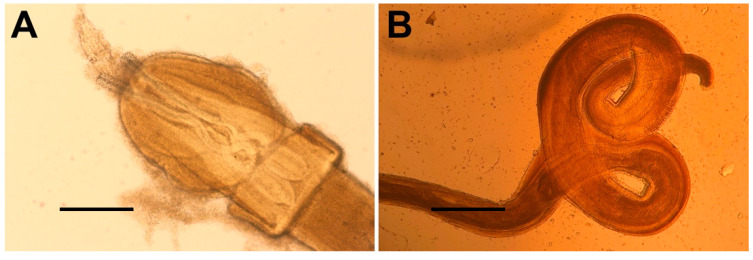
Gastrointestinal parasites found in *S. canicula* in the present study. (**A**) *Nybelinia* sp. (Few, 1926), scale bar 0.75 mm; (**B**) *Proleptus obtusus* (Dujardin, 1845), scale bar 0.5 mm.

In total, 29 parasites were found only in the gastro-intestinal tract: eight in the stomach, 20 in the spiral valve, and one in the liver. No parasites were found in the other anatomical districts. Only 18 of 75 sharks were parasitized (24%). All parasites were identified to the lowest possible taxonomic level. Of the parasites found, 27 (of which seven in the stomach, 19 in the spiral valve, and one in the liver) belonged to the phylum Platyhelminthes, class Cestoda, order Trypanorhyncha, family Tentaculariidae, and to the genus *Nybelinia* sp. (Few, 1926) (Figure 4A) and two (one in the stomach and one in the spiral valve) belonged to the phylum Nematoda, class Chromadorea, order Rhabditida, family Physalopteridae, and to the species *Proleptus obtusus* (Dujardin, 1845) (Figure 4B). All nematodes were found in the stomach and spiral valve and belonged to a single shark specimen.

To analyze epidemiological indices of prevalence (P%), average abundance (AA), and average intensity (AI) for the 75 sharks collected, two sub-tables were created (Table 2): in the first, the prevalence index (P%) was calculated for each sampling month for both sexes; in the second table, the AA and the AI indices were calculated for all the parasite species found (29) in all the months and for all the sharks collected, 18 of which were parasitized.

The highest prevalence of parasitized sharks was found in May 2018 for both males (25%) and females (41.10%) and, subsequently, in February 2017 (6.66% and 14.28% for females and males respectively). In January 2018, only females were parasitized (38.80%). In August, no samples were found parasitized. No differences were observed between sexes in the accumulation of parasites (*p* value = 0.66; *n* = 18).

### 3.2. Hematology

**Figure 5:**
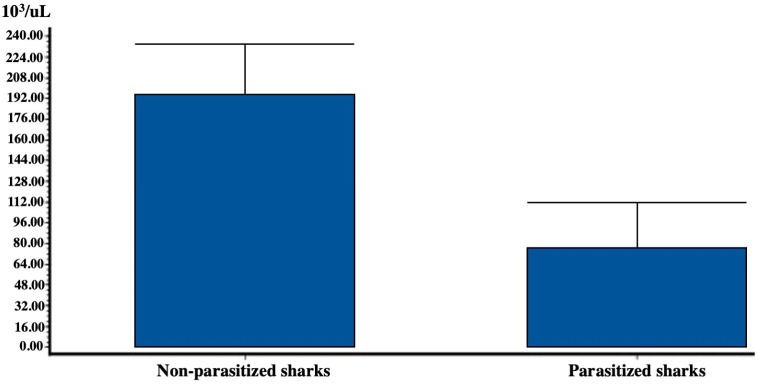
Difference ± sd between leukocyte count (10^3^/μL) and presence/absence of parasites (N of non-parasitized sharks = 25; N of parasitized sharks = 7).

We obtained the leukocyte count for all 32 blood samples collected, 25 of which belonged to not parasitized sharks and seven to parasitized ones, while the RBC and the other hematological parameters were carried out only for ten not parasitized samples. The average values of the blood count analysis are shown in the following table (Table 3):

The correlations between the components examined in complete blood counts did not provide significant results. However, the correlation between shark weight and leukocyte count (R^2^ = 0.01; *p* value = 0.02; *n* = 32) was found to be statistically significant, expressed by an inverse proportion. Leukocyte count was also compared to the presence/absence of parasites in sharks whose leukocyte values were considered, and the results of these values are reported in Figure 5.

Parasitized sharks showed a much lower average number of leukocytes than non-parasitized ones. This difference was considered extremely significant (U = 3; U′ = 172, *p* value < 0.0001).

### 3.3. Ecotoxicology

Results on trace element concentration found at point A and C of the vertebrae are presented in Table 4.

For 12 specimens, trace element concentrations found in the vertebrae at point C were then compared with those found in the skin and liver by means of the Kruskal–Wallis test: As and Mn were significantly more accumulated in the vertebrae, Ni and Fe in the skin, and Cd in the liver (Table 5).

The concentrations of all elements, expressed in descending order, present in the target tissues were as follows:

Vertebrae: As > Mn > Zn > Fe > Ni > Cu > Pb > Cd

Skin: Fe > Zn > As > Cu > Mn > Ni > Pb > Cd

Liver: Fe > Zn > As > Cu > Mn > Ni > Cd > Pb

Regarding the analysis of trace elements in the vertebrae, the eight element concentrations considered were related to sex, TL, two different stages of the biological cycle of the sharks, and to the parasitic load using linear regressions and the Mann–Whitney test to verify significant differences and relationships. Significant results relating to non-essential trace elements (Pb, As, Cd) that could be toxic even at low concentration were as follows:

- in vertebrae, Pb decreased with the TL of the specimens (R = 0.8; *p* value = 0.02; *n* = 75);
- in vertebrae, As decreased from the juvenile (Point A of the vertebrae) to the adult (Point C of the vertebrae) phase (U = 2083; U′ = 3542; *p* value = 0.006; *n* = 75).

Arsenic was also more concentrated in point C of the vertebrae of the 18 parasitized sharks than in the 57 non-parasitized ones (U = 277; U′ = 749; *p* value = 0.003; *n* = 75). This was the only trace element that showed significant differences with parasitic load.

In vertebrae, Cd did not show significant correlations and differences with sex, TL, parasitic load, nor with the two stages of the biological cycle. Results relating to the essential and partially essential trace elements were as follows:

- in vertebrae, Mn increased from the juvenile (Point A of the vertebrae) to the adult (Point C of the vertebrae) phase (U = 1168.5; U′ = 4456.5; *p* value < 0.0001; *n* = 75);
- in vertebrae, Zn increased from the juvenile (Point A of the vertebrae) to the adult (Point C of the vertebrae) phase (U = 1447; U′ = 4178; *p* value < 0.0001; *n* = 75);
- in vertebrae, Ni decreased from the juvenile (Point A of the vertebrae) to the adult (Point C of the vertebrae) phase (U = 2281; U′ = 3344; *p* value = 0.04; *n* = 75);
- in vertebrae, Cu and Fe did not show significant correlations or differences with sex, TL, parasitic load, nor with the two stages of the biological cycle.

## 4. Discussion

All 75 specimens of *S. canicula* were collected in the central Tyrrhenian Sea at 150 m depth were. Since 1948, the temperature trend, according to the study of Painter and Tsimplis [28], remained around 0 °C during all seasons, not influencing the parameters examined in this research.

Only 29 parasites were found in the gastro-intestinal tract of the 75 sampled sharks, belonging to two unique taxa: 27 cestodes belonged to the genus *Nybelinia* and two nematodes belonged to the species *Proleptus obtusus*. As pointed out by Dallarès et al. [4], the population of *S. canicula* in the north-western Mediterranean is characterized by an impoverished helminthic community, with little richness and diversity but strongly dominated by a single species. The same was also observed in this research, where the dominant parasite was represented by the cestode genus *Nybelinia*. The low average richness, as well as the diversity and high dominance of the infra-communities observed for *S. canicula*, have also been reported for other small sharks from different areas: according to Isbert et al. [29], the infra-communities of *Etmopterus spinax* (Linnaeus, 1758) of the North Atlantic were also characterized by low average richness and high values of dominance. Moore [6] and Henderson et al. [30] reported a low number of helminthic infra-communities in the populations of *S. canicula* and *Squalus acanthias* (Linnaeus, 1758) of the North Atlantic; Henderson et al. [30] recovered ten parasites from *Squalus acanthias*, and in few larger but always small sharks, Palm et al. [31] reported six parasites of *Heptranchias perlo* (Bonnaterre, 1788), three of *Deania calcea* (Lowe, 1839), seven of *Deania profundorum* (Smith & Radcliff, 1912), and nine of *Deania histricosa* (Garman, 1906). Isbert et al. [29] therefore suggested that a poor helminthic community, with low richness and diversity but with high dominance values, could represent a characteristic of small-sized sharks. In addition, several studies on the helminthic communities of teleosts have also reported a lower richness of parasites in the Mediterranean compared to Atlantic populations [32]. The reduced size of *S. canicula*, the reduced consumption of benthic organisms, and the lower biomass and abundance of animal communities in the Mediterranean can explain its modest helminthic fauna. The maximum size of *S. canicula* in the Mediterranean is smaller than the Atlantic one, and a similar pattern has been observed for Mediterranean specimens of *Galeus melastomus* (Rafinesque, 1810) compared to their Atlantic counterparts [33,34]. The prevalence of parasites in the gastro-intestinal tract of the sharks showed similar values in January and May 2018 for females (38.80% vs. 41.10% respectively), while for males, no parasites were observed during winter (0%), and 25% of sharks were parasitized during spring. On the contrary, a drastic reduction of prevalence was observed for both females (6.66%) and males (14.28%) in February 2017, while no parasites were observed for both sexes in August 2017 (0%). This discrepancy among the prevalence values is due to the low number of sharks collected, especially in August (only six sharks collected) and to a low number of parasitized specimens (only 18 sharks of 75 collected), not allowing us to observe the presence of a seasonal helminthic populations pattern, since helminthic dynamics are mostly influenced both by the feeding habits of the hosts, which largely depend on the availability of the intermediate hosts in their bathymetric distribution and by the dynamics and biological characteristics of the host population [35,36].

### 4.1. The Role of Trace Elements in Weakening of the Immune System

This study represented the first contribution on the hematology of *S. canicula*. The relationship between the number of leukocytes in the 32 sharks analyzed and their weights was extremely significant and can be described by an inverse proportionality: the heavier, and therefore larger sharks had a lower number of leukocytes and vice versa. In adult humans, for example, the number of white blood cells is much lower than that present in infants (4000–1000 10^3^/μL and 25,000–10,000 10^3^/μL, respectively) [37]. However, studies of this type relating to elasmobranchs are not present in the literature, and therefore, more sampling is necessary to justify these claims. Parasitized sharks should present a greater number of leukocytes in order to defend their organism from pathogens and, therefore, from parasites. However, we observed that the number of leukocytes and the presence of parasites was inversely proportional, since parasitized sharks (7) had a much lower number of leukocytes than non-parasitized ones (25). The decrease in leukocytes proportional to the parasitic load contracted by organisms has been observed by several authors, linked to the immunosuppression by accumulation of some trace elements in animals’ tissues: Priyadarshani et al. [38] observed a decrease in the immune response of the Indian green frog *Euphlyctis hexadactylus* (Lesson, 1834) due to the accumulation of trace elements (As, Cu, Zn, Pb, Cd) in contaminated urban environments, compared to unpolluted natural ones. Increasing concentrations of some trace elements could therefore be related to the decrease in the blood of leukocytes and splenocytes [24], to a change in neutrophils in relation to lymphocytes, to a change in total Immunoglobulins levels, but also in the spleen weight in relation to body weight and phagocytic activity of peritoneal macrophages, splenocytes, and leukocytes compared to the counterparts of the reference site. These components of the immune system are essential to protect animals from helminthic infections and their decrease, caused by the accumulation of trace elements, could lead to an increased risks of parasite infections. Other studies conducted with natural exposure or oral administration of trace elements [39,40] showed that increasing concentrations of some metals (Hg, Cu, Pb, Cd, Zn) increased the number of the monoclonal parasite nematodes *Ascaris suum* (Goeze, 1782) in guinea pigs *Cavia porcellus* (Pallas, 1766), as the result of a considerable suppression of lymphocytes T and B and of the phagocytic ability of macrophages during the migration phase of *Ascaris suum*, when compared to a group of infected animals that remained untreated. Studies in different taxa such as *Barbus barbus* [41], *Oreochromis hybrid* [42], and *Larus hyperboreus* [43] showed the same relationship between concentrations of trace elements and the degree of helminthic infestation. Therefore, as with other vertebrates, trace element bioaccumulation in sharks could also have a similar immunosuppressive role, which leads to an increase in the degree of infestation, as observed in this research for arsenic in *S. canicula* in the Mediterranean Sea, but we need more data on leukocytes count and parasitic load to demonstrate that.

### 4.2. Trace Elements Accumulation in S. canicula from Tyrrhenian Sea

This study represented the first contribution to the analysis of trace elements in the vertebrae of *S. canicula*.

#### 4.2.1. Lead (PB)

The anomalies of Pb (but also of As, Cu, and Zn) in the Tuscan waters are linked to the iron and steel industry of Piombino, and in the soils of southern Tuscany, high values exceeding 138 ppm are reported [44]. The maximum concentrations of Pb were recorded in Piombino, in the south of Elba, in the Gulf of Follonica, and in the Argentario, places close to the sampling area of *S. canicula* specimens [44]. Lead was more accumulated in the vertebrae (but the difference with other tissues was not significant) since it comes from the diet [45] and, in the same tissue, it decreased significantly with the increase in the TL of the samples. The negative correlations between TL and trace elements (except for Hg) are well known in the literature [16,46,47] since the accumulation of trace elements is the result of the difference between the concentration of elements in organisms and their purification over time. In this regard, Jeffree et al. [48] observed in chondrichthyans that embryos can accumulate Pb in eggs quickly if exposed, even for a short time, to this trace element, and its concentration increases with increasing growth and development of the egg. In the first stages of embryonic development, the organism implements some defense mechanisms against pathogens, but defensive actions against potential toxic elements are not yet known [48]. However, after the sharks are born, in addition to the metabolic changes in the diet between the juvenile and adult phases the rectal gland plays a detoxifying role [12]. It is therefore probable that the lesser spotted dogfish accumulates Pb both during its development in the egg and during its juvenile phase but, when it reaches sexual maturity and changes diet, it succeeds in expelling it.

#### 4.2.2. Arsenic (As)

The concentration of As along the Tuscany coast where the sharks were collected is quite high: both in Piombino and in the south of Elba Island, peaks of 166 ppm have also been recorded [44]. In Scarlino, there is also a high rate of As pollution, linked to mixed sulfide hydrothermal mineralization, and concentrations of up to 1000 ppm can also be reached. The causes of this pollution would be due to the floods of the Pecora River, rich in insoluble arsenic salts deposited in the clay sediments and to industrial activities for processing pyrite [44]. Arsenic was significantly more concentrated in the vertebrae since it comes from the diet [45]. However, it is not clear why, in vertebrae, absorption from the juvenile to adult stages can significantly decrease. In previous studies, trace elements were analyzed in several organs such as the liver and muscle of different demersal and pelagic sharks in several locations in the Mediterranean [45,49]: Storelli et al. [45] stated that the relationship between different stages of the biological cycle and accumulation of As in the muscle of *S. canicula* and *Galeus melastomus* was positive, while Storelli et al. [49] stated that the relationship between different stages of the biological cycle and accumulation of As in the liver of the same species was negative. Furthermore, arsenic is not subject to biomagnification, but to metabolization and detoxification in the liver of sharks as observed by Kaise and Fukui [50] who observed that the liver contains more As than the muscles and suggested that there is a detoxification process (methylation) by the squalene that takes place in the liver of adult specimens: the inorganic As would be transported to the liver, methylated, and accumulated, and this also occurs for *S. canicula* when the concentration of the toxic trace element reaches 1%.

#### 4.2.3. Cadmium (Cd)

Cd pollution in Tuscany derives from sphalerite deposits associated with zinc, and the maximum concentration allowed is 1.5 μg/L for marine waters, while for sediments, this value is 0.3 ppm [44]. Cadmium was significantly more concentrated in the liver than in other organs since it comes from the diet. According to Marcovecchio et al. [51], this metal accumulates in large concentrations in the liver of sharks and dolphins and increases with the increase in animal size. The concentration of essential metals, such as Zn and Cu, in the liver and muscles of fish and marine mammals tends to decrease with growth (probably in juveniles, these essential metals are required for rapid growth), while after sexual maturity, it increases with the increase in Cd [51]. As suggested by the authors, the high concentrations of Zn are related to the presence of Cd, as Zn exerts a protective effect against Cd toxicity in the liver: the increase in Cd induces the synthesis of metallothionein proteins (MT) in the liver to which Cd will bind together with Zn and Cu, detoxifying the organism [51].

#### 4.2.4. Other Trace Elements (Mn, Zn, Ni, Cu, Fe)

Manganese was significantly more concentrated in the vertebrae, and it is an essential micronutrient and a fundamental cofactor for many enzymes, as well as supporting metabolism, protein production, cellular processes, and activation of reproductive hormones [52]. Diet represents the main route for the absorption of Mn in elasmobranchs: in a comparative study on the accumulation of Mn radionuclides in a teleost and in an elasmobranch, Pentreath [53] concluded that the absorption of Mn radioisotopes exclusively from the water was insufficient to explain the internal concentrations of Mn radionuclide. More recently, Mathews and Fisher [54] have experimentally determined that more than 90% of Mn accumulated in the soft tissues of the lesser spotted dogfish *S. canicula* was derived from food sources. Furthermore, the increase in Mn from the juvenile to the adult phase probably occurs during the nutrition shift, since the adult lesser spotted dogfish diet is composed of small fishes in which there is a greater concentration of proteins able to bind to Mn [52]. Zinc was more accumulated in the liver even if the difference in accumulation was not significant: Zn is a fundamental metal for a wide range of physiological processes, including cell growth, neurotransmission, and signaling, as well as playing a vital role in the production, structuring and maintenance of proteins [55]. Diet represents the primary source of Zn intake both in elasmobranchs and teleosts [54], also in agreement with the results of this study and the dietary intake of Zn in *S. canicula* is similar to that estimated for other fish. Similar to Mn, our data are in accordance with the works of Mathews et al. [52] and Mathews and Fisher [54] in which elasmobranchs accumulated high concentrations of Zn during diet shift from juvenile to adult phase, showing an increase in the concentration of this element in the adult phase, probably due to the greater predation of small fishes in which there is a higher concentration of proteins able to bind to Zn [56]. Nickel was significantly more concentrated in the skin, as also noted by De Boeck et al. [12]. Results indicated a significant decrease in this metal from the juvenile to the adult phase in vertebrae. In the work of Vas [57], the ability to accumulate Ni is greater in pelagic sharks (e.g., *Prionace glauca*) that live far from the coastal area and have a piscivorous diet. Since adults of *S. canicula* feed on small fishes, an increase in Ni from the juvenile to the adult phase should be expected. However, it should not be excluded that Ni is transmitted directly from mother to the embryo through eggs, and this could justify a greater amount of Ni in juveniles and its discrimination over time by renal activity [12], a hypothesis that can only be confirmed by chemical analysis of embryos and eggs. Copper was more concentrated in the liver than in the other tissues, although this difference was not significant. Cu in the liver, in association with Zn and Cd, is excreted during the adult phase of sharks by the metallothionein proteins [51]. Iron was significantly more concentrated in the skin and is an essential metal for metabolic processes [58]. In the study of De Boeck et al. [12], Fe showed a high concentration in the skin of *S. canicula*. The motivation is to be found in the affinity between placoid scales and iron [48,54]: placoid scales are composed of collagen, glycosaminoglycans and apatite, which bind metal cations.

## 5. Conclusions

The analysis performed has contributed to increasing knowledge on the parasitology, hematology, and ecotoxicology of one of the most abundant benthopelagic shark species in the Mediterranean. The fact that the parasitic load in this shark samples from the Tyrrhenian Sea is low and is characterized by a low richness and diversity may probably be linked to the small size of this species, to the low consumption of benthic organisms, and to the scarce biomass and abundance of animal communities in the Mediterranean. Some elements such as Pb, As, Cd, Mn, Cu, and Zn could probably be accumulated through the diet in the vertebrae and liver; others such as Ni and Fe could come from environmental pollution through the skin. It would seem that that marine pollution in the central Tyrrhenian Sea has reached critical levels that should make us reflect, and it is important to reiterate that the survival of humans is linked to the balance and health of marine ecosystems. It is therefore essential to promote plans of monitoring and protection for elasmobranchs and the environment, both locally and extended to the entire Mediterranean basin.

## Figures and Tables

**Table 1:** Biometric parameters of sampled *S. canicula* specimens.

	Total *±* sd	Females *±* sd	Males *±* sd
**TL (cm)**	39.65 ± 3.01	39.77 ± 2.80	39.34 ± 3.40
**W (g)**	234.51 ± 74.10	239.49 ± 77.20	219.8 ± 6.74

**Table 2:** Epidemiological indices (upper: prevalence index P% divided for sampling periods and sexes; lower: average abundance (AA) and average intensity (AI) indexes for all 29 parasites found in all the months and sharks collected, 18 of which were parasitized).

Months	N° of Examined Hosts		N° of Parasitized Hosts		P% Males	P% Females
	Males	Females	Males	Females		
Feb 17	7	15	1	1	14.28	6.66
Aug 17	1	5	0	0	0.00	0.00
Jan 18	4	18	0	7	0.00	38.80
May 18	8	17	2	7	25.00	41.10
**Total**	**20**	**55**	**3**	**15**	**15.00**	**27.70**

AA: 0.38, AI: 1.61.

**Table 3:** Blood analysis of the 32 *S. canicula* samples, 25 of which were not parasitized and 7 parasitized (RBC: red blood cells; HGB: hemoglobin; MCV: mean corpuscular volume; PLT: platelets; WBC: white blood cells).

	RBC (10^6^/μL) *n* = 10	HGB (g/dL) *n* = 10	MCV (fL) *n* = 10	PLT (10^3^/μL) *n* = 10	WBC (10^3^/μL) *n* = 32
**Average value**	0.028	4.680	127.330	50.900	213.790
**SD (standard deviation)**	0.012	0.590	24.128	13.634	26.460
***n* (number of samples)**	10	10	10	10	10
**SEM (standard error)**	0.004	0.180	7.630	4.310	8.360

**Table 4:** Trace element concentrations (μg/g) at point A and C of the vertebrae expressed as mean ± sd.

Trace Element	Vertebrae (Point A) ± sd *n* = 75	Vertebrae (Point C) ± sd *n* = 75
Pb	8.03 ± 0.40	5.20 ± 0.80
As	384 ± 37.00	192 ± 62.00
Mn	69.70 ± 5.01	136 ± 75.00
Ni	12.03 ± 1.25	9.50 ± 1.40
Fe	140 ± 7.00	96 ± 12.00
Cu	18 ± 0.50	7 ± 1.50
Cd	0.06 ± 0.02	0.09 ± 0.01
Zn	60.90 ± 4.30	125.70 ± 34.00

**Table 5:** Trace element concentrations (ppm) in target tissues expressed as mean ± sd; KW: Kruskal–Wallis value; n.s.: not significant; * significant (significance at 10%), *** extremely significant (significance at 1%).

Trace Element	Vertebrae (Point C) *±* sd *n* = 12	Skin *±* sd *n* = 12	Liver *±* sd *n* = 12	KW *±* sd *n* = 12	*p* Value
Pb	5.20 ± 0.80	0.60 ± 0.08	0.50 ± 0.01	1.15	0.5 n.s.
As	192 ± 62	48 ± 12.20	104 ± 34	6.03	0.049 *
Mn	136 ± 75	12 ± 1.40	6 ± 2.90	30.50	<0.0001 ***
Ni	9.50 ± 1.40	11 ± 0.70	1.50 ± 0.09	13.51	<0.0001 ***
Fe	96 ± 12	592 ± 110	288 ± 99.90	21.55	<0.0001 ***
Cu	7 ± 1.50	3.40 ± 2.70	11 ± 2.30	1.63	0.44 n.s.
Cd	0.09 ± 0.01	0.14 ± 0.02	0.81 ± 0.10	22.12	<0.0001 ***
Zn	125.70 ± 34	72 ± 13	138.30 ± 27	15.53	0.5 n.s.

## Data Availability

Not applicable.
